# Supplementary material for: Threshold analyses on combinations of testing, population size, and vaccine coverage for COVID-19 control in a university setting
Source: PLoS One. 2021 Aug 9;16(8):e0255864. doi: 10.1371/journal.pone.0255864 (PMC8351932; doi:10.1371/journal.pone.0255864)
Supplement: S1 Text — (DOCX) [file pone.0255864.s007.docx]

**S1 Text. APPENDIX:**

**Threshold analyses on combinations of testing, population size, and vaccine coverage for COVID-19 control in a university setting**

Xinmeng Zhao^⁋1^, Hanisha Tatapudi^⁋1^, George Corey^2^, and *Chaitra Gopalappa^1^

^1^Department of Mechanical and Industrial Engineering, University of Massachusetts Amherst, MA, USA

^2^ University Health Services, University of Massachusetts Amherst, MA, USA

*Corresponding author:

e-mail: [chaitrag@umass.edu](mailto:chaitrag@umass.edu)

^⁋^ These authors contributed equally to this work

**S1A Table. Epidemic parameters used for the compartmental simulation model**

| **Parameter** | **Description**  (notations in main paper Methods) | **Age Range** | **Median** | **Range (LB)** | **Range (UB)** | **Source** |
| --- | --- | --- | --- | --- | --- | --- |
| Baseline transmission rate | $p$ |  | 14.3 | 11.6 | 17 | [1–4] |
| Latent period duration (days) | Not infectious; not symptomatic ($days_{L}$) |  | 2.52 |  |  | [5] |
| Infectious incubation period (days) | Infectious and not symptomatic ($days_{incub}-days_{L}$) |  | 3.5 |  |  | [6,7] |
| Incubation period duration (days) | $days_{incub}$ |  | 5.4 |  |  | [6,7] |
| Proportion of cases that never show symptoms | $prop_{asymp}$ |  | 0 |  |  | [7] |
| Time from onset of symptoms to recovery (days) | $days_{IR}$ |  | 7 |  |  | [7] |
| Base testing rate | $a_{B}$ (Rate for generating 65% symptom-based diagnosis) |  | 0.2 |  |  | [6–8] |
| Time from symptoms to hospitalization (days) | $days_{Q_{I}H}$ |  | 3.5 |  |  | [9] |
| Time from diagnosis to recovery (days) | $days_{Q_{I}R}$(same as $days_{Q_{I}H}$) |  | 3.5 |  |  | [9] |
| Proportion of cases that are hospitalized (also used as proxy for proportion of cases that are severe) | $prop_{hosp}$ (same for $prop_{severe}$) | 0–19 | 2% | 1.6–2.5 |  | [10] |
|  |  | 20–44 | 14% | 14.3–20.8 |  |  |
|  |  | 45–54 | 24% | 21.2–28.3 |  |  |
|  |  | 55–64 | 25% | 20.5–30.1 |  |  |
|  |  | 65–74 | 35% | 28.6–43.5 |  |  |
|  |  | 75–84 | 44% | 30.5–58.7 |  |  |
|  |  | ≥85 | 32% | 31.3–70.3 |  |  |
|  |  | Total | 12% | 20.7–31.4 |  |  |
| Duration of hospitalization for those who recover | $days_{HR}$ | 0-9 | 2 |  |  | [11] |
|  |  | 10-19 | 1.8 |  |  |  |
|  |  | 20-29 | 2.5 |  |  |  |
|  |  | 30-39 | 3.7 |  |  |  |
|  |  | 40-49 | 3.9 |  |  |  |
|  |  | 50-59 | 3.8 |  |  |  |
|  |  | 60-69 | 4.3 |  |  |  |
|  |  | 70-79 | 4.6 |  |  |  |
|  |  | 80-89 | 4.4 |  |  |  |
|  |  | 90-100 | 4.8 |  |  |  |
|  |  | Overall | 3.9 | 2.4 |  |  |
| Proportion of cases that recover | $prop_{recover}$ |  | Male;Female | Both |  | [11,12] |
|  |  | 0-9 | 100%;100% | 98% |  |  |
|  |  | 10-19 | 100%;100% | 97% |  |  |
|  |  | 20-29 | 93%;98% | 96% |  |  |
|  |  | 30-39 | 95%;98% | 93% |  |  |
|  |  | 40-49 | 92%; 97% | 89% |  |  |
|  |  | 50-59 | 88%; 93% | 82% |  |  |
|  |  | 60-69 | 81%; 88% | 70% |  |  |
|  |  | 70-79 | 64%; 73% | 51% |  |  |
|  |  | 80-89 | 39%; 52% | 12% |  |  |
|  |  | 90-100 | 36%; 54% | 12% |  |  |
| Duration of hospitalization for deaths | $days_{HD}$ | 0-9 | 2 |  |  | [11] |
|  |  | 10-19 | 1.8 |  |  |  |
|  |  | 20-29 | 4 |  |  |  |
|  |  | 30-39 | 2.8 |  |  |  |
|  |  | 40-49 | 5.6 |  |  |  |
|  |  | 50-59 | 5.9 |  |  |  |
|  |  | 60-69 | 5.7 |  |  |  |
|  |  | 70-79 | 5 |  |  |  |
|  |  | 80-89 | 3.9 |  |  |  |
|  |  | 90-100 | 3 |  |  |  |
|  |  | Overall | 4.8 | 2.3 |  |  |
|  |  |  |  |  |  |  |

**S1B Table. Transmission rate estimates; Baseline values from [1–4], and relative risks from [2];**

|  | **Relative risk [2]** | | | **Lower (baseline)** | | | **Median (baseline)** | | | **Upper (baseline)** | | |
| --- | --- | --- | --- | --- | --- | --- | --- | --- | --- | --- | --- | --- |
|  |  |  |  | **11.60%** | | | **14.00%** | | | **11.60%** | | |
| **Intervention** | Lower | Median | Upper | Lower | Median | Upper | Lower | Median | Upper | Lower | Median | Upper |
| **Facemask** | 0.40 | 0.56 | 0.79 | 4.6% | 6.5% | 9.2% | 5.6% | 7.8% | 11.1% | 6.8% | 9.5% | 13.4% |
| **3ft distancing** | 0.26 | 0.49 | 0.93 | 1.5% | 2.8% | 5.3% | 1.8% | 3.4% | 6.4% | 2.2% | 4.1% | 7.7% |
| **6ft distancing** | 0.10 | 0.20 | 0.41 | 1.2% | 2.3% | 4.8% | 1.4% | 2.8% | 5.7% | 1.7% | 3.4% | 7.0% |

**S1C Table. Literature review of population behavior for number of contacts and compliance to face mask usage and social distancing for university and non-university setting (includes information from pre-pandemic and during pandemic surveys and model assumptions)**

| **Reference** | **Type of study** | **Timeline** | **Location/setting** | **Summary for number of contacts** | **Summary of face mask and social distancing compliance** |
| --- | --- | --- | --- | --- | --- |
| *University setting* | | | | | |
| [13] | Survey of students on the likelihood to engage in COVID-19 disease mitigation behaviors | During pandemic in early August, 2020 | United States/Kansas State University |  | 70.06% participants responded definitely will and 20.82% responded possibly will for usage of face mask in public spaces. |
|  |  |  |  |  | 78.2% participants responded definitely will and 16.3% responded probably will for use of face mask in classrooms. |
|  |  |  |  |  | 48.95% participants responded definitely will and 38.86% responded possibly will for observing 6 ft social distancing in public spaces. |
|  |  |  |  |  | 47.39% participants responded definitely will and 41.22% responded probably will for observing 6 ft social distancing in classrooms. |
| [14] | Survey of students regarding COVID related experiences | During early pandemic between April 25–30, 2020 | United States/University students across the country | Average number of contacts is 12.7. | Approximately 50.8% participants reported always wearing a face mask or covering in public. |
|  |  |  |  | Contacts ranged from 0 up to 100 |  |
|  |  |  |  | 4.2% were uncertain about number of contacts. |  |
| [15] | Modelling study to determine whether in-person instruction is safe to continue during the pandemic | During pandemic | United States/University setting | The model generates an average of 11 traceable and 8 nontraceable contacts per person per day when there are face-to-face classes and social distancing is not being exercised. | The transmission probabilities in the study were reduced by 50% in lieu of implementing non-pharmacological interventions (like mask wearing). |
| [16] | Survey conducted to determine the spread and frequency of protective behaviors, emotional and anxiety status. | During pandemic in early April, 2020 | Turkey/University students across the country |  | 50% reported wearing protective gloves and masks. |
| [17] | Survey conducted to understand social distancing and contact behavior. | Before pandemic between March and May 2003 | Belgium/University setting | The number of conversational contacts is 18.1 on weekdays and 12.3 on weekends. |  |
| [18] | Survey conducted to understand social distancing and contact behavior. | Before pandemic between 2003 and 2006 | Germany/University setting | The median number of conversational contacts varied between 6 and 11 for weekdays and weekends, respectively. |  |
| [19] | Survey conducted to understand social distancing and contact behavior. | Before pandemic between June and July 2004 | Germany/School setting | A mean number of contacts per day for children was 25.1. |  |
|  |  |  |  | The mean number of contacts per day for adults was 7.5. |  |
| [20] | Online survey of contacts, behavior, and symptoms | During pandemic between September and November, 2020 | United Kingdom/University of Bristol (UoB) | The median number of contacts on the day before the interview was 2. | Residential students were asked not to host non-residents in their household. |
|  |  |  |  | The interquartile range was between 1 and 5; the mode was 1, and the mean was 6.1. | Residential students were allowed to meet others outside of their household. |
|  |  |  |  | Among all participants, 8% had 20 contacts. | Students were asked to conform to government suggested social distancing guidelines and other mitigation measures (like face coverings) |
|  |  |  |  | Among students, 57% of student contacts were other UoB students/staff. |  |
|  |  |  |  | The mean number of contacts reported by students for the previous day was 6.1, with a median of 2. |  |
| [21] | Modeling study that uses data of online survey of contacts, behavior, and symptoms | During pandemic but contact data obtained from pre-pandemic survey conducted in September 2010 | United Kingdom/University of Bristol (UoB) | Students were assumed to living in groups with a maximum of 24 individuals. | The model reduced transmission probability by 25% for face covering and 50% for social distancing. |
|  |  |  |  | Intervention measures would reduce group size (i.e., number of students that share bathroom/kitchen facilities) from 24 to 20 or 14 students was seen as the least effective intervention. |  |
| [22] | Online questionnaire survey to determine knowledge, attitudes, and practices towards COVID-19 | During pandemic between May and June, 2020 | Japan/University students across the country |  | Approximately 52.1% of university students in Jordan and 98.0% of university students in China reported wearing a facemask when leaving home. |
|  |  |  |  |  | 86.9% of undergraduate students from Indonesia and 96.4% of Japanese students wore masks frequently in a crowded place. |
|  |  |  |  |  | The frequency of handwashing and mask-wearing were reported at a median of 96.4% |
| *Non-university setting* | | | | | |
| [23] | Survey for individual's perceptions about risk and behavior related to COVID-19 | During pandemic between May and June, 2020 | United Stated (six states in the U.S.: Colorado, Iowa, Louisiana, Massachusetts, Michigan, Washington)/Non-university setting |  | 66% participants reported always wearing a mask in public indoor spaces. |
|  |  |  |  |  | 46% participants reported always physically distancing outside home. |
| [24] | Multiple surveys conducted about COVID behavior | During pandemic between April and November 2020. | United States/Non-university setting |  | 77% participants reported to closely adhering to recommendations to wearing masks in November. |
| [25] | Survey to assess the impact of physical distancing policies on contacts | During pandemic between March and September, 2020 | United States/Non-university setting | A median of 2 contacts (0 non-household) in Wave 0, a median of 3 contacts (1 non-household) in Wave 1, a median of 3 contacts (1 non-household) in Wave 2, and a median of 4 contacts (2 non-household) in Wave 3. |  |
| [26] | Survey conducted to understand social distancing and contact behavior. | During early pandemic between March and April 2020 | United States/Non-university setting | Individuals who completely or mostly isolate have 5 contacts per day. |  |
|  |  |  |  | Individuals who don't attempt to isolate themselves have an average of 52 contacts. |  |
|  |  |  |  | Working adults have an average of 13.9 contacts and non-working adults have an average of 4 contacts. |  |
| [27] | Large-scale survey of social encounters | Pre-pandemic survey conducted in October 2010 | Great Britain/Non-university setting | According to the survey, individual-only contacts peaked at a maximum of 20. |  |
|  |  |  |  | The distribution of contacts was characterized by a lognormal body with a power-law tail and an exponent of 22.45 |  |
| [28] | Large-scale survey of social contacts | Pre-pandemic survey conducted in September 2010 | Great Britain/Non-university setting | The mean number of individual contacts is 7.97. |  |
|  |  |  |  | Mean total number of contacts is 26.75. |  |
|  |  |  |  | When adjusted for age and gender biases, mean individual contacts and total contact rise slightly to 8.28 and 28.50. |  |
| [29] | Population-based survey to assess mixing patterns in eight European countries | Pre-pandemic survey conducted between May 2005 and September 2006 | 8 European counties (Belgium, Germany, Finland, Great Britain, Italy, Luxembourg, Netherlands, and Poland)/Non-university setting | Mean contacts per person per day is 13.4. |  |
|  |  |  |  | German participants reported fewest daily number of contacts with a mean of 7.95 and Italians reported the highest number with a mean of 19.77. |  |
|  |  |  |  | 23%, 21%, 14%, 3%, and 16% of the reported contacts are made at home, at work, at school, while travelling, and during leisure activities, respectively when contacts were pooled together. |  |
| [30] | Survey conducted to evaluate the impact of government interventions on social contact patterns. | During pandemic between March and June, 2021 | Luxembourg/Non-university setting | Luxembourg, Italian, Belgian, British, and German residents reported and average of 17.5, 19.8, 11.8, 11.7, and 8 social contacts per day respectively, before the pandemic. | The average number of contacts per day as reported by participants was 7.1 contacts after the lockdown. |
|  |  |  |  | After the lockdown participants reported an average of 7.1 contacts per day on. | Participants reported that 61.7% of the total contacts occurred without a facemask, which is a mean of 4.9 contacts. |

References

1. Zhao PJ. A Social Network Model of the COVID-19 Pandemic. Epidemiology; 2020 Mar. doi:10.1101/2020.03.23.20041798

2. Chu DK, Akl EA, Duda S, Solo K, Yaacoub S, Schünemann HJ, et al. Physical distancing, face masks, and eye protection to prevent person-to-person transmission of SARS-CoV-2 and COVID-19: a systematic review and meta-analysis. The Lancet. 2020;395: 1973–1987. doi:10.1016/S0140-6736(20)31142-9

3. Wan H, Cui J-A, Yang G-J. Risk estimation and prediction of the transmission of coronavirus disease-2019 (COVID-19) in the mainland of China excluding Hubei province. Infect Dis Poverty. 2020;9: 116. doi:10.1186/s40249-020-00683-6

4. He S, Tang S, Rong L, 1 School of Mathematics and Information Science, Shaanxi Normal University, Xi’an, 710119, China, 2 Department of Mathematics, University of Florida, Gainesville, 32611, USA. A discrete stochastic model of the COVID-19 outbreak: Forecast and control. Mathematical Biosciences and Engineering. 2020;17: 2792–2804. doi:10.3934/mbe.2020153

5. Ma S, Zhang J, Zeng M, Yun Q, Guo W, Zheng Y, et al. Epidemiological parameters of coronavirus disease 2019: a pooled analysis of publicly reported individual data of 1155 cases from seven countries. Infectious Diseases (except HIV/AIDS); 2020 Mar. doi:10.1101/2020.03.21.20040329

6. Li R, Pei S, Chen B, Song Y, Zhang T, Yang W, et al. Substantial undocumented infection facilitates the rapid dissemination of novel coronavirus (SARS-CoV-2). Science. 2020;368: 489–493. doi:10.1126/science.abb3221

7. Hill A, Levy M, Xie S, Sheen J, Shinnick J, Gheorghe A, et al. Modeling COVID-19 Spread vs Healthcare Capacit. 27 May 2020. Available: https://alhill.shinyapps.io/COVID19seir/

8. Wu Z, McGoogan JM. Characteristics of and Important Lessons From the Coronavirus Disease 2019 (COVID-19) Outbreak in China: Summary of a Report of 72 314 Cases From the Chinese Center for Disease Control and Prevention. JAMA. 2020;323: 1239. doi:10.1001/jama.2020.2648

9. MIDAS 2019 Novel Coronavirus Repository. [cited 24 Apr 2020]. Available: https://github.com/midas-network/COVID-19

10. CDC COVID-19 Response Team, CDC COVID-19 Response Team, Bialek S, Boundy E, Bowen V, Chow N, et al. Severe Outcomes Among Patients with Coronavirus Disease 2019 (COVID-19) — United States, February 12–March 16, 2020. MMWR Morb Mortal Wkly Rep. 2020;69: 343–346. doi:10.15585/mmwr.mm6912e2

11. Richardson S, Hirsch JS, Narasimhan M, Crawford JM, McGinn T, Davidson KW, et al. Presenting Characteristics, Comorbidities, and Outcomes Among 5700 Patients Hospitalized With COVID-19 in the New York City Area. JAMA. 2020;323: 2052. doi:10.1001/jama.2020.6775

12. Stokes EK, Zambrano LD, Anderson KN, Marder EP, Raz KM, El Burai Felix S, et al. Coronavirus Disease 2019 Case Surveillance — United States, January 22–May 30, 2020. MMWR Morb Mortal Wkly Rep. 2020;69: 759–765. doi:10.15585/mmwr.mm6924e2

13. COVID-19 Mitigation Behavior Survey Results. In: Kansas State University [Internet]. [cited 26 Apr 2020]. Available: https://www.k-state.edu/covid-19/communications/every-wildcat-a-wellcat/fall-2020-mitigation-behavior-survey-results.html

14. Cohen AK, Hoyt LT, Dull B. A Descriptive Study of COVID-19–Related Experiences and Perspectives of a National Sample of College Students in Spring 2020. Journal of Adolescent Health. 2020;67: 369–375. doi:10.1016/j.jadohealth.2020.06.009

15. Gressman PT, Peck JR. Simulating COVID-19 in a University Environment. Mathematical Biosciences. 2020;328: 108436. doi:10.1016/j.mbs.2020.108436

16. Akdeniz G, Kavakci M, Gozugok M, Yalcinkaya S, Kucukay A, Sahutogullari B. A Survey of Attitudes, Anxiety Status, and Protective Behaviors of the University Students During the COVID-19 Outbreak in Turkey. Front Psychiatry. 2020;11: 695. doi:10.3389/fpsyt.2020.00695

17. Beutels P, Shkedy Z, Aerts M, Van Damme P. Social mixing patterns for transmission models of close contact infections: exploring self-evaluation and diary-based data collection through a web-based interface. Epidemiol Infect. 2006;134: 1158–1166. doi:10.1017/S0950268806006418

18. Mikolajczyk RT, Kretzschmar M. Collecting social contact data in the context of disease transmission: Prospective and retrospective study designs. Social Networks. 2008;30: 127–135. doi:10.1016/j.socnet.2007.09.002

19. Mikolajczyk RT, Akmatov MK, Rastin S, Kretzschmar M. Social contacts of school children and the transmission of respiratory-spread pathogens. Epidemiol Infect. 2008;136: 813–822. doi:10.1017/S0950268807009181

20. Nixon E, Trickey A, Christensen H, Finn A, Thomas A, Relton C, et al. Contacts and behaviours of university students during the COVID-19 pandemic at the start of the 2020/21 academic year. Public and Global Health; 2020 Dec. doi:10.1101/2020.12.09.20246421

21. Brooks-Pollock E, Christensen H, Trickey A, Hemani G, Nixon E, Thomas A, et al. High COVID-19 transmission potential associated with re-opening universities can be mitigated with layered interventions. Infectious Diseases (except HIV/AIDS); 2020 Sep. doi:10.1101/2020.09.10.20189696

22. Hatabu A, Mao X, Zhou Y, Kawashita N, Wen Z, Ueda M, et al. Knowledge, attitudes, and practices toward COVID-19 among university students in Japan and associated factors: An online cross-sectional survey. Zeeb H, editor. PLoS ONE. 2020;15: e0244350. doi:10.1371/journal.pone.0244350

23. COVID-19 Reopening Survey Data. In: Risk and Social Policy Group [Internet]. [cited 26 Apr 2020]. Available: https://static1.squarespace.com/static/5ec4464f22cd13186530a36f/t/5ef0e413c14aa311f58404fe/1592845331607/FINAL_onepager_wave1.pdf

24. Lazer D, Santillana M, Perlis R, Quintana A, Ognyanova K, Green J, et al. THE COVID STATES PROJECT: A 50-STATE COVID-19 SURVEY REPORT #26: TRAJECTORY OF COVID-19-RELATED BEHAVIORS. In: www.covidstates.org [Internet]. Nov 2020 [cited 7 May 2021]. Available: https://news.northeastern.edu/wp-content/uploads/2020/11/COVID19-CONSORTIUM-REPORT-26-BEHAVIORS-NOV-2020.pdf

25. Feehan DM, Mahmud AS. Quantifying population contact patterns in the United States during the COVID-19 pandemic. Nat Commun. 2021;12: 893. doi:10.1038/s41467-021-20990-2

26. Rothwell J. Americans’ Social Contacts During the COVID-19 Pandemic. In: GALLUP [Internet]. 21 Apr 2020 [cited 7 May 2021]. Available: https://news.gallup.com/opinion/gallup/308444/americans-social-contacts-during-covid-pandemic.aspx

27. Danon L, House TA, Read JM, Keeling MJ. Social encounter networks: collective properties and disease transmission. J R Soc Interface. 2012;9: 2826–2833. doi:10.1098/rsif.2012.0357

28. Danon L, Read JM, House TA, Vernon MC, Keeling MJ. Social encounter networks: characterizing Great Britain. Proc R Soc B. 2013;280: 20131037. doi:10.1098/rspb.2013.1037

29. Mossong J, Hens N, Jit M, Beutels P, Auranen K, Mikolajczyk R, et al. Social Contacts and Mixing Patterns Relevant to the Spread of Infectious Diseases. Riley S, editor. PLoS Med. 2008;5: e74. doi:10.1371/journal.pmed.0050074

30. Latsuzbaia A, Herold M, Bertemes J-P, Mossong J. Evolving social contact patterns during the COVID-19 crisis in Luxembourg. Shaman J, editor. PLoS ONE. 2020;15: e0237128. doi:10.1371/journal.pone.0237128

31. Ritchie H, Ortiz-Ospina E, Beltekian D, Mathieu E, Hasell J, Macdonald B, et al. Mortality Risk of COVID-19. In: Our World in Data [Internet]. [cited 5 May 2020]. Available: https://ourworldindata.org/mortality-risk-covid#the-case-fatality-rate

32. CDC. COVID-19 Pandemic Planning Scenarios. In: Centers for Disease Control and Prevention [Internet]. [cited 5 May 2021]. Available: https://www.cdc.gov/coronavirus/2019-ncov/hcp/planning-scenarios-archive/planning-scenarios-2020-05-20.pdf

33. Cashore J, Duan N, Janmohamed A, Wan J, Zhang Y, Henderson S. COVID-19 Mathematical Modeling for Cornell’s Fall Semester. 2020. Available: https://cpb-us-w2.wpmucdn.com/sites.coecis.cornell.edu/dist/3/341/files/2020/10/COVID_19_Modeling_Jun15-VD.pdf
